# Plant Secondary Metabolites as Defense Tools against Herbivores for Sustainable Crop Protection

**DOI:** 10.3390/ijms23052690

**Published:** 2022-02-28

**Authors:** Pratap Adinath Divekar, Srinivasa Narayana, Bhupendra Adinath Divekar, Rajeev Kumar, Basana Gowda Gadratagi, Aishwarya Ray, Achuit Kumar Singh, Vijaya Rani, Vikas Singh, Akhilesh Kumar Singh, Amit Kumar, Rudra Pratap Singh, Radhe Shyam Meena, Tusar Kanti Behera

**Affiliations:** 1 Indian Council of Agricultural Research-Indian Institute of Vegetable Research (IIVR), Varanasi 221305, India; rajeev09150@gmail.com (R.K.); achuits@gmail.com (A.K.S.); ranivijaya78@gmail.com (V.R.); tusar@rediffmail.com (T.K.B.); 2 Institute of Agricultural Sciences, Banaras Hindu University, Varanasi 221305, India; srinivasa@bhu.ac.in (S.N.); radheento@gmail.com (R.S.M.); 3 IPL Biologicals Ltd., Gurugram 122003, India; bhupendraento@gmail.com; 4 Indian Council of Agricultural Research-National Rice Research Institute, Cuttack 753006, India; basanagowda.g@icar.gov.in; 5 Indira Gandhi Krishi Vishwavidyalaya, Raipur 492012, India; aishwaryaray01@gmail.com; 6 Indian Council of Agricultural Research-Indian Institute of Vegetable Research, Regional Research Station, Sargatia, Kushinagar 274406, India; vikaschf@gmail.com; 7 College of Horticulture, Banda University of Agriculture and Technology, Banda 210001, India; dr.akhileshento@rediffmail.com; 8 Rajmata Vijayaraje Scindia Krishi Vishwa Vidyalaya, Sheopur 476339, India; dramit_w9@rediffmail.com; 9 Acharya Narendra Deva University of Agriculture and Technology, Ayodhya, Krishi Vigyan Kendra, Kotwa, Azamgarh 276207, India; rudrapsingh.doe@gmail.com

**Keywords:** secondary metabolites, insect herbivores, defense regulation, phytohormones, insect adaptations, natural enemy, pollinators, sustainable protection

## Abstract

Plants have evolved several adaptive strategies through physiological changes in response to herbivore attacks. Plant secondary metabolites (PSMs) are synthesized to provide defensive functions and regulate defense signaling pathways to safeguard plants against herbivores. Herbivore injury initiates complex reactions which ultimately lead to synthesis and accumulation of PSMs. The biosynthesis of these metabolites is regulated by the interplay of signaling molecules comprising phytohormones. Plant volatile metabolites are released upon herbivore attack and are capable of directly inducing or priming hormonal defense signaling pathways. Secondary metabolites enable plants to quickly detect herbivore attacks and respond in a timely way in a rapidly changing scenario of pest and environment. Several studies have suggested that the potential for adaptation and/or resistance by insect herbivores to secondary metabolites is limited. These metabolites cause direct toxicity to insect pests, stimulate antixenosis mechanisms in plants to insect herbivores, and, by recruiting herbivore natural enemies, indirectly protect the plants. Herbivores adapt to secondary metabolites by the up/down regulation of sensory genes, and sequestration or detoxification of toxic metabolites. PSMs modulate multi-trophic interactions involving host plants, herbivores, natural enemies and pollinators. Although the role of secondary metabolites in plant-pollinator interplay has been little explored, several reports suggest that both plants and pollinators are mutually benefited. Molecular insights into the regulatory proteins and genes involved in the biosynthesis of secondary metabolites will pave the way for the metabolic engineering of biosynthetic pathway intermediates for improving plant tolerance to herbivores. This review throws light on the role of PSMs in modulating multi-trophic interactions, contributing to the knowledge of plant-herbivore interactions to enable their management in an eco-friendly and sustainable manner.

## 1. Introduction

All living organisms have to face environmental and biotic challenges during their lifetime. According to Darwin’s evolutionary theory, “survival of the fittest” or “natural selection” enables the fittest organism to compete, survive and reproduce. The fittest organisms have the diverse genetic potential to defend themselves, or resist or avoid stress consequences which hamper their physiological functions, permitting them to grow, develop and survive. This adaptive evolution ensures the ecological specialization of a species for a specific niche [1] and ultimately results in speciation [2]. Being sessile organisms, plants are continuously exposed to various biotic and abiotic stresses, such as herbivore or pathogen attack, drought, salinity, UV-irradiation, extreme temperatures, and nutritional imbalances in natural environments [3,4,5]. Phytophagous herbivores are said to be responsible for destroying one-fifth of the world’s total crop production annually [6]. 

Plants have evolved several types of secondary metabolites as a defensive shield to protect themselves from phytophagous herbivores [7]. Nearly 200,000 PSMs have been isolated and characterized which is a small number relative to the 391,000 described plant species [8]. Upon exposure to herbivores, secondary metabolites accumulate at an increased level and act as signaling molecules to upregulate the defense response genes. PSMs ensure the competitiveness and survival of plants under stress conditions. Recently, the signaling role of these metabolites in defending plants has received more attention [5]. PSMs include alkaloids, terpenes, amines, glucosinolates, cyanogenic glucosides, quinones, phenolics, peptides and polyacetylenes [9]. PSMs have no role in the basic life processes of plants, but they play a vital role in adaptation and defense against herbivores. PSMs are synthesized through several metabolites and intermediates that are engaged in plant defense. These pathways start from primary metabolic pathways, which are the ultimate precursors of PSMs. Primary metabolites are actively engaged in the regulation of the normal growth and development of plants. However, secondary metabolites are only involved in plant defense against herbivores. Although the role of these plant metabolites is different, they are interlinked, as primary metabolites act as precursors for the synthesis of secondary metabolites [10]. Primary and secondary metabolites are different in their structure, function and distribution in different tissues of plants. 

Plants have devised a sophisticated recognition and signaling system which ensures early recognition of herbivore attacks and triggers a powerful defense response [11]. Recent genetic and chemical investigations have demonstrated the multifunctional nature of PSMs, which act as potent regulators of plant growth, defense and primary metabolism. Induced plant defenses are driven by the phytohormones jasmonic acid (JA) and salicylic acid (SA), and the associated pathways interact in a complicated fashion at the transcript and protein level. Adverse effects on the survival of chewing insects (*Heliothis virescens*) and sucking insects (*Myzus persicae*) were reported after JA and SA application [12]. Secondary metabolites may also have hormone-like properties by binding to specific receptor proteins [13]. There is a synergistic effect of PSMs operating together to tackle herbivore damage [14]. A combination of these secondary metabolites is likely to prevent or delay the development of resistance by insect herbivores [15]. However, insects have been found to show different adaptive responses, including detoxification, excretion or sequestration of plant secondary metabolites [10]. Although the role of secondary metabolites in plant defense is well established, in addition some metabolites are used to attract insect pollinators and parasitoids [16,17]. Secondary metabolites are seen as not only a cost-effective and ecologically friendly means to sustain agriculture, but they also compete with agrochemicals in terms of plant growth and protection [18].

This review presents the latest updated information regarding the protective role of secondary metabolites in sustainably maintaining plant health. Furthermore, we highlight the role of phytohormones in regulating secondary metabolite biosynthesis and plant defense signaling. Overall, this review provides substantial evidence that secondary metabolites derived from plants can be used to develop effective, environmentally friendly, and cost-effective integrated strategies aimed at increasing crop growth and yield and thus ensuring sustainable agriculture.

## 2. Types of Secondary Plant Metabolites

**Figure 1:**
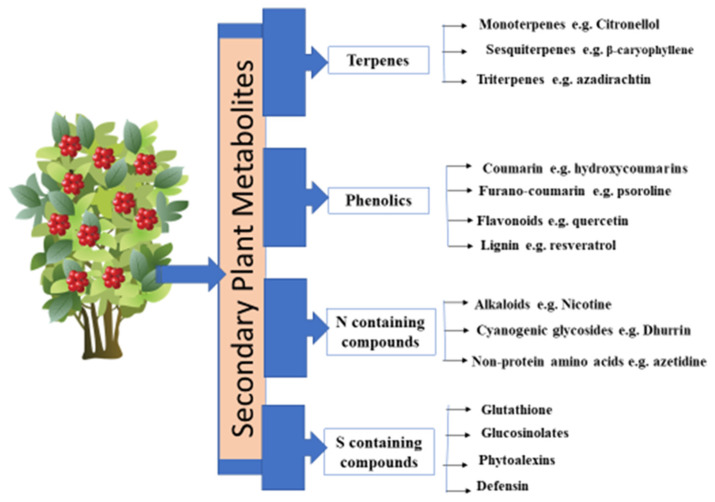
Types of secondary metabolites.

PSMs constitute a major defensive weapon of plants to defend themselves against a broad range of phytophagous herbivores, so it is necessary to investigate the biosynthesis and the application of these metabolites to deploy them as an eco-friendly and sustainable pest management option. Recent analytical tools and techniques have enabled elucidation of the role of secondary metabolites in plant defense [19]. These metabolites are categorized into four different groups: terpenoids, phenolics, and nitrogen and sulfur-containing compounds (Figure 1).

### 2.1. Terpenes

Terpenes are the largest group of plant secondary metabolites. Although most terpenes are important in plant defense, some terpenes (e.g., gibberellins, brassinosteroids) are involved in primary functions, such as plant growth and development. Terpenes comprise around 25,000 compounds [20], with diverse functions including feeding deterrence, direct toxicity, or oviposition deterrence. Specialist herbivores can tolerate terpenoids and utilize them as an attractant to locate their host plants and as feeding stimulants [10]. Terpenes can serve as attractants to pollinating insects [20]. Terpenes indirectly protect plants by increasing the efficacy of herbivore natural enemies through the release of specific volatiles [21]. Some examples of terpenes that play an active role in plant defense are iridoids, benzoxazinoids, and volatile compounds, such as mono and sesquiterpenes, α-bisabolene and β-caryophyllene [22].

### 2.2. Phenolic Compounds

Plant phenols are a heterogeneous group of secondary metabolites which include nearly 10,000 compounds. Phenolics are the most widely distributed secondary metabolites that comprise a hydroxyl functional group (phenyl group) on an aromatic ring. Phenolics are diverse compounds based on chemical structure and comprise simple phenols (e.g., catechols and hydroxybenzoic acid derivatives), flavonoids, catechol melanins, stilbenes, condensed tannins, and lignins. These metabolites are actively engaged in protecting plants against herbivores and attracting pollinators. Phenolics can directly act as toxins to herbivores or can be oxidized by peroxidases or polyphenol oxidases to toxic metabolites which cause physiological disturbances in insect growth and developmental processes [23]. 

### 2.3. Sulfur-Containing Plant Secondary Metabolites

Sulfur-containing secondary metabolites are glucosides mainly reported from the Brassicaceae and Capparales plant taxons. Glucosides are a derivative of amino acids and around 120 molecular structures have been reported [24]. The amino acid precursor from the side chain typically determines the type of glucoside. A higher concentration of glucosinolate was reported in younger leaves and reproductive portions of plants. Four different groups of glucosides include metabolites derived from methionine (aliphatic glucosinolates), glucosinolates derived from tryptophan (indole glucosinolates), glucosinolates derived from tyrosine or phenylalanine (aromatic glucosinolates), and glucosinolates derived from different amino acids or one unknown amino acid [24]. Glucosinolates are typically present in cell vacuoles [16] and are covered by myrosinases (thioglucosidase). Herbivore injury results in disruption of plant cells, as a result of which glucosinolates are broken down by myrosinases into toxic metabolites, such as nitriles, thiocyanates and isothiocyanates. These breakdown products of glucosinolates are as effective as synthetic insecticides [25] and have been shown to be extremely toxic to herbivorous insects and to repel them from feeding [26]. 

### 2.4. Nitrogen-Containing Compounds

Nitrogen-containing secondary metabolites include alkaloids. To date, around 10,000 different derivatives of alkaloids have been reported across the plant kingdom [27]. Alkaloids are divided into three groups on the basis of biosynthesis [28]:

(a) true alkaloids (e.g., nicotine, morphine, quinine and atropine);
(b) pseudo-alkaloids (e.g., capsaicin, solanidine and caffeine); and
(c) proto-alkaloids (e.g., yohimbine, mescaline and hordenine).

Both true alkaloids and proto-alkaloids are derived from amino acids; however, pseudo-alkaloids are not produced from amino acids. True alkaloids are obtained from amino acids and they share a nitrogen-containing heterocyclic ring. They are highly reactive in nature and have potent biological activity. They form water-soluble salts by conjugating with acids, and many of them are crystalline in nature. Almost all true alkaloids are bitter in taste and solid, except nicotine, which is a brown liquid [29]. Proto-alkaloids contain a nitrogen atom, which is derived from an amino acid but is not part of the heterocyclic ring system. L-tryptophan and L-tyrosine are the main precursors of this type of alkaloid. This minor group is structurally composed of simple alkaloids. Yohimbine, mescaline, and hordenine are the main alkaloids of this type [28]. Alkaloids are reported from gymnosperms, angiosperms and primitive genera of plants [19]. Alkaloid toxicity to herbivores is due to disruption of neuronal signal transduction, and interference in DNA replication, protein synthesis and enzyme activity [30].

## 3. Understanding the Biosynthesis of Secondary Plant Metabolites 

**Figure 2:**
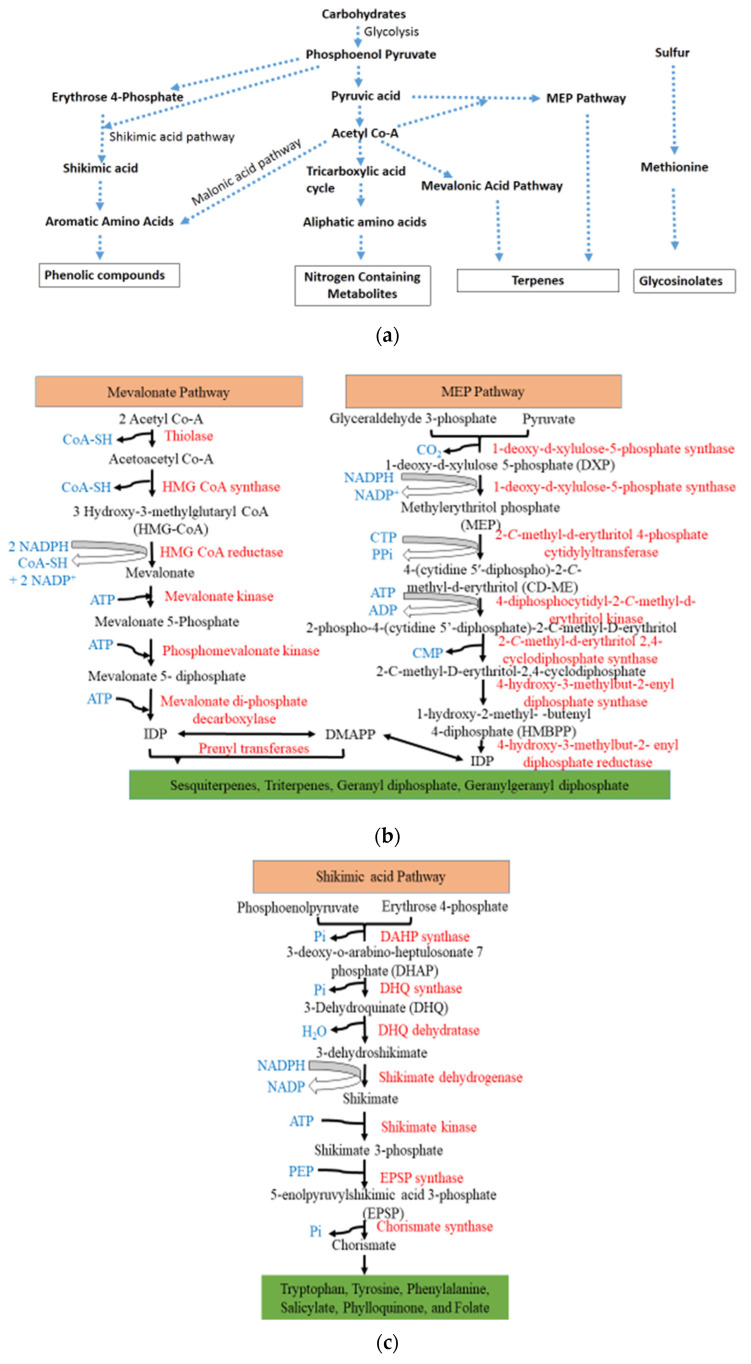
(**a**) Biosynthesis pathways of phenolic, nitrogen and sulfur containing compounds. (**b**) Terpenoid biosynthesis pathway in plants. (**c**) Phenolic biosynthesis pathway in plants.

Biosynthesis and signaling pathways of PSMs are complex and these metabolites act in combination or synergistically against specific stresses through different modes of action to prevent the resistance or tolerance to these phytochemicals [31]. Specific pathways and substrate-specific enzymes play a key role in the biosynthesis of PSMs. Several precursors from primary metabolites, such as amino acids, fatty acids, sugars or acetyl CoA, are involved in PSM biosynthesis. These precursor molecules are the by-products of the tricarboxylic acid cycle and shikimate pathways (Figure 2a). As primary metabolites are distributed in every plant tissue, their biosynthetic pathways are conserved in plants. The maintenance of these metabolic pathways has resulted in a restricted basic metabolic framework. Metabolic processes, including frequent glycosylation, methylation, hydroxylation, acylation, oxidation, phosphorylation, and prenylation, as well as chemical alterations due to tailoring of enzymes, have led to a wide range of modifications in basic structures [31].

PSMs and their respective biosynthetic pathways:

i. phenolic compounds (shikimate pathway),
ii. terpenes (mevalonic or methylerythritol phosphate pathway), and
iii. nitrogen and sulfur containing compounds (tricarboxylic acid cycle pathway) [9].

PSM biosynthesis is a highly complex, but coordinated, process that comprises metabolon formation and channeling of metabolites. The channeling of metabolites occurs in different plant cells and cellular compartments which ensures specific metabolite synthesis in a particular cell or tissue and prevents metabolic interventions [32].

The synthesis of PSM is organ, tissue, cell and development stage-specific indicating that a chain of specific transcription factors is involved to activate and transcribe the genes of secondary metabolites in plants. Transcription factors are involved in the regulation of PSM biosynthesis, transport and storage. Most of the biosynthetic pathways occur in the cytoplasm, though some alkaloids (e.g., coniine, caffeine), furanocoumarins and a few terpenes (e.g., monoterpenes, diterpenes, phytol and carotenoids) are synthesized in the chloroplast [33]. Sterols, sesquiterpenes and dolichols are synthesized in the endoplasmic reticulum (ER) or the cytosol [31]. 

Terpenes are synthesized from isopentenyl diphosphate (IPP) and its isomer dimethylallyl diphosphate (DMAPP). These building blocks are produced via two distinct pathways: the acetyl-CoA-derived cytosolic mevalonate (MVA) pathway and the pyruvate-derived plastidial 2-C-methyl-D-erythritol-4-phosphate (MEP) pathway (Figure 2b, [34]) They are classified as monoterpenes (C10), with two isoprene units, sesquiterpenes (C15), with three isoprene units, diterpenes (C20), with four isoprene units, triterpenes (C30), with six isoprene units and tetraterpenes (C40), with eight isoprene units. Terpenes serve as essential components of various phytohormones, pigments and sterols. They also serve as allelochemicals, defensive toxins and herbivore deterrents [35].

Shikimic acid is the precursor of the shikimate pathway and is produced from a combination of erythrose 4-phosphate (pentose phosphate pathway) and phosphoenolpyruvate (glycolytic pathway). The amino acids phenylalanine, tyrosine and tryptophan are the products of the shikimate pathway, and serve as precursors of phenolics and nitrogen-containing secondary metabolites [36]. Phenylalanine is a precursor for phenolics, such as flavonoids, lignin, condensed tannins, betalain pigments and quinones, whereas tryptophan is a precursor of alkaloids, phytoalexins and indole glucosinolates. Chorismate is the end-product produced through several steps in the shikimate pathway (Figure 2c). Chorismite is a precursor of tryptophan, tyrosine, phenylalanine, salicylate, phylloquinone, and folate in higher plants and is regulated by enzymes such as chorismate mutase, iso-chorismate synthase, anthranilate synthase, and aminodeoxychorismate synthase [37]. PSM storage in plants sometimes exceeds more than 10% of total dry weight. Water-soluble PSMs are stored in the vacuole (epidermal cells). Lipophilic PSMs are not sequestered in vacuoles but are released into raisin ducts, oil cells, trichomes, or cuticles [31].

The regulation of different biosynthetic pathways, and the particular enzymes involved, is achieved by gene duplication and adaptations to the specific stress conditions. Several studies have demonstrated that different plants evolved independently, i.e., they show convergent evolution where these plants can synthesize similar compounds or structurally different metabolites [38]. Therefore, the occurrence and distribution of a particular PSM within plants is neither ubiquitous nor does it follow a clear phylogenetic pattern [19].

The complex cascades involved in the synthesis of PSMs are still a subject of further research. A thorough understanding of the biosynthesis of PSMs will enable us to manipulate, or intervene in, the process of plant defense regulation. 

## 4. Secondary Metabolite Functional Role in the Regulation of Plant Defense and Early Detection of Herbivore Attack

Plants generally increase their resistance and decrease their growth in response to herbivore attacks. A phytohormonal signaling network enables this prioritization. The rice transcription factor WRKY70, which is activated by herbivore-induced mitogen-activated protein kinase signaling, plays a critical role in prioritizing defense over growth by positively regulating jasmonic acid (JA) and negatively regulating gibberellin (GA) levels in response to attack by the chewing herbivore *Chilo suppressalis*. Proteinase inhibitors are activated and resistance to *C. suppressalis* is achieved through WRKY70-dependent JA biosynthesis. WRKY70 induction, on the other hand, makes rice plants more susceptible to the rice brown planthopper, *Nilaparvata lugens*. Studies with GA-deficient rice lines demonstrated that WRKY70-dependent GA signaling is responsible for susceptibility to *N. lugens*. Thus, prioritizing defense over growth results in a resistance trade-off, which has significant consequences for plant defense regulation [39]. Plants regulate defense activation to save metabolic energy and avoid self-damage. Feedback regulation, which includes both positive and negative feedback loops embedded into early defense signals, is often used to titrate defense investment [39] and hormonal networks [40]. These feedback loops have the drawback of not providing direct information on the final level of defensive activation (i.e., the production of defense metabolites per se). Herbivores can interfere with the synthesis of defense chemicals at many levels, including the last steps of biosynthesis [41], so incorporating them directly into regulatory feedback loops should help plants effectively monitor and regulate defense. Secondary metabolites, as defense activation readouts, may also assist plants in maximizing synergy between several defenses and compensating for failures in the defense pathway.

Because many secondary metabolites are compartmentalized and/or retained in inactive forms, decompartmentalization and/or activation may aid plants in detecting herbivore tissue damage [31]. Plants that have been damaged perceive a range of endogenous chemicals as danger signals, which are referred to as damage-associated molecular patterns (DAMPs). Secondary metabolites would be employed as DAMPs. Secondary metabolites that are also DAMPs include green-leaf volatiles [42].

Plant defenses can also be regulated by volatile compounds, such as terpenoids, green-leaf volatiles, and aromatic chemicals, in addition to glucosinolates and benzoxazinoids [43]. Most of these volatiles are generated in response to herbivore attacks, and they can directly induce or prime hormonal defense signaling pathways and resistance. In maize, mutants that cannot synthesize volatile indole are unable to condition their systemic tissues to release terpenes quickly in response to herbivore attacks. Adding indole to the headspace of maize plants restores this priming phenotype [44]. Rice (*Oryza sativa*) plants also respond to indole through priming of early defense signaling elements, such as the map kinase OsMPK3. Transgenic plants lacking the OsMPK3 gene are no longer responsive to indole, implying that indole functions by activating early defensive signaling [45]. Five types of secondary metabolites (i.e., glucosinolates, benzoxazinoids, terpenes, aromatics, and green-leaf volatiles) have now been demonstrated to serve as potential plant defense regulators. It is exciting to think that there are potentially several other secondary metabolites with similar regulatory functions.

Plants have evolved efficient defense systems to protect themselves from herbivores. They can recognize and respond to invaders by activating defense-related signaling pathways, such as mitogen-activated protein kinase (MAPK) and hormonal signaling, which results in the expression of several defense-related genes and phytochemicals [46]. Induced resistance to herbivores is mediated primarily through jasmonic acid (JA), salicylic acid (SA), and ethylene (ET)-mediated signaling [47]. Herbivore-plant interaction is a two-way process, i.e., plants activate a specific defense response after insect injury to protect themselves. Plants must be able to distinguish between physical injury and insect feeding to activate an insect-specific defense. The oral secretion or oviposition fluid of insects has been found to contain specific active substances known as elicitors. These are recognized by plants and are important in the formation of defense-related downstream signaling cascades [48]. Oral secretions have also been observed to suppress plant defense mechanisms [49]. Elicitors are divided into six categories based on their chemical structure and composition: enzymes, fatty acid amino acid conjugates (FACs), fatty acids, peptides, esters, and benzyl cyanide [50]. Enzymes, fatty acids, FACs, and peptides are secreted in oral secretions, while esters and benzyl cyanide are released in oviposition fluids during egg-laying [48,51,52]. Different types of elicitors may have different effects and modes of action.

An enzyme elicitor, β-glucosidase, from *Pieris brassicae* regurgitates was the first insect elicitor to be recognized and reported [53]. The plant defense is activated and a series of volatiles are released by β-glucosidase in cabbage, lima beans, and corn plants [26,54]. These released volatiles attract the parasitoid *Cotesia glomerata*. Thus, the β-glucosidase in *P. brassicae* induces indirect plant defense [53]. Sucking insects also express this type of elicitor, in addition to chewing herbivores. β-glucosidase is predominantly present in *Nilaparvata lugens*, which raises the concentrations of jasmonic acid (JA), hydrogen peroxide (H_2_O_2_), and ethylene [55]. This leads to multiple downstream signaling cascades and the release of volatiles, such as dodecenal and tetradecane, which in turn attracts the parasite *Anagrus nilaparvatae* [55]. β-glucosidases are compartmentalized away from the inactive, glucosidically bound volatiles, i.e., glucosinolates. A mixture of these two elements leads to β-glucosidase activity, releasing volatiles, such as isothiocyanate, thiocyanate, and nitriles, as a result of insect feeding [49,56].

Plants recognize certain elicitors from insect oral secretions (OS) which enter through wounds during insect feeding in plant tissue and trigger a chain of interlinked signaling pathways involved in the synthesis of defensive metabolites. Mitogen-activated protein kinases (MAPKs) are ubiquitously present in all eukaryotic organisms and are actively engaged in many cellular processes of normal growth and stress responses. Insect OS activates the two types of MAPKs, salicylic acid-induced protein kinase (SIPK) and wound-induced protein kinase (WIPK) in plants. MAPKs are important in active regulation of the insect herbivore-induced dynamics of phytohormones, jasmonic acid, ethylene, and salicylic acid. MAPKs are also required for transcriptional activation of herbivore defense-related genes and accumulation of defensive metabolites [11]. Induced defense is maintained by signaling processes that regulate the downstream responses to insect-herbivore specific cues, including transcriptional activation of genes that encode enzymes involved in the synthesis of defense metabolites [57]. The activation of MAPKs, which modulate phytohormone levels and restructure the transcriptome and proteome in preparing for plant defense, is the most crucial process in signaling after insect attack.

Herbivore-associated molecular patterns (HAMPs) include all herbivore-induced signaling metabolites recognized by the host plants and, thus, elicit defense responses [58]. Insect OS-containing elicitors are the most explored HAMPs in understanding herbivore-plant interactions [47]. These elicitors activate insect-specific responses when they enter into plant tissue during insect feeding. The first elicitor identified in the armyworm *Spodoptera exigua* oral secretions was N-(17-hydroxy linolenoyl)-l-glutamine (volicitin). Volicitin can induce volatiles in maize to attract parasitoids of *S. exigua* [59]. Fatty acid-amino acid conjugates (FACs), structurally similar to volicitin, were reported from OS *Manduca sexta* larvae [60]. Application of FACs to wounds, mimicking caterpillar feeding, activates insect-specific responses in plants, including the enhanced concentration of jasmonic acid (JA), ethylene (ET) and salicylic acid (SA) and reshaping of the transcriptome [61]. FACs are elicitors that activate MAPK signaling [57]. Focused research is needed to understand how elicitors activate defense signaling (e.g., MAPKs) and downregulate defense reactions in plants. In view of the complexity of insect-herbivore interactions, there is potential for the existence of several insect-derived elicitors, which may trigger insect-specific defense response in a particular plant species.

## 5. Role of Phytohormones in Regulation of Induced Plant Defense through PSMs

Phytochemicals and protein-based defenses enable plants to detect attacking organisms and to inhibit them prior to extensive loss [62]. Phytohormones elicit defense responses to herbivore attacks in addition to their role in growth, development and physiological processes of the plant [63]. JA is considered to be an important hormone responsible for the activation of direct and indirect defense responses to herbivores [64]. When herbivores damage plants, linoleic acid released from plants is converted to active jasmonate via the octadecanoid pathway mediated by various enzymes, including lipoxygenase (LOX), and finally activates resistance mechanisms and defense-related gene expression [65]. The chewing of plant parts by herbivores causes deoxygenation of linoleic acid and linolenic acid by LOX to hydroperoxy derivatives which are converted to 12-oxo-phytodienoic acid (12-OPDA) by allene oxide synthase (AOS) and allene oxide cyclase (AOC) in plastids. Further, reactions take place in the peroxisome, i.e., reduction of OPDA to JA by OPDA reductase 3 (OPR 3) [66]. Induction of lipoxygenase activity following insect infestation has been recorded in various crops against major pests [67] and plants deficient in LOX are susceptible to insect pests [68]. JASMONATE ZIM-domain (JAZ) proteins bind to transcription factors, i.e., MYC2, to restrict JA signal output [69]. However, JA levels will be at a lower level in the absence of stress but will increase rapidly when any stress condition, such as wounding or herbivory attack, occurs [46].

Salicylic acid (SA) is another ubiquitous key signaling molecule in plants derived from benzoic acid [70]. Reports suggest there are two pathways by which SA is synthesized in plants. Plants synthesize SA from cinnamate by the activity of phenylalanine ammonia-lyase (PAL) while other reports suggest the bulk of SA is produced from isochorismate [71]. However, both pathways utilize the common product chorismite (end-product of the shikimate pathway) to produce SA [72]. The salicyclic acid pathway is activated in plants in response to piercing and sucking insects [73]. The puncturing of a leaf by leaf minor adults activates the JA pathway, while larval mining activates both JA and SA defense pathways [74].

Piercing and sucking types of insects induce ethylene production in plants [75]. Ethylene emission and JA accumulation in plants upon feeding herbivory have been reported [76]. The ethylene pathway does not work in an isolated manner to elicit a defense response but works with a network of plant hormones [74]. Multiple signaling cascades are involved in the induction of plant defenses by insect feeding. Ethylene signaling, for example, makes *Arabidopsis* more vulnerable to the generalist herbivore, Egyptian cotton worm (*Spodoptera littoralis*). In comparison to wild-type plants, the hookless1 mutation, which affects a downstream component of ethylene signaling, conferred Egyptian cotton worm resistance. Similarly, ein2, a mutation in a key component of the ethylene signaling system, resulted in Egyptian cotton worm resistance that was comparable to hookless 1 [77]. Phytohormones do not act independently—there is a complex signaling network that interacts together. The crosstalk between JA and SA signaling pathways leads to antagonistic [78] and synergistic reactions [79]. Endogenous or external application of phytohormones induces production of secondary metabolites to defend plants against herbivores (Table 1). Other phytohormones are also induced in response to herbivore attacks, such as abscisic acid (ABA), auxin, gibberellins and cytokinins [46]. ABA levels were found to increase in plants following herbivore attacks [80]. ABA synthesis in plants synergizes with JA-driven defense responses in plants against herbivores [81]. Cytokinin-related genes are strongly regulated in *Nicotiana attenuata* following fatty acid-amino acid conjugate (FAC) elicitation suggesting a role of cytokinins in the hormonal regulatory network [82]. Cytokinin priming in plants increased the levels of JA, linolenic acid, wound inducible transcripts and reduced weight gain in the larvae of the gypsy moth, *Lymantria dispar* [83]. *Manduca sexta* attack on *Nicotiana attenuata* leads to increased levels of IAA in plants locally [84] and is followed systemically by the production of JA-dependent secondary metabolites (anthocyanins and phenolamides in the stems) suggesting the involvement of IAA in plant defense communication against herbivores in association with JA [84]. Gibberellins are naturally occurring phytohormones; gibberellic acid (GA) was the first structurally characterized gibberellin [85]. GA downregulated poplar plants have shown moderate resistance to insect pests [86]. A thorough understanding of the role of phytohormones will enable us to impart greater resistance in plants against herbivores by regulating the production of PSMs.

## 6. Effect of Plant Secondary Metabolites on the Physiology and Behavior of Herbivores

**Figure 3:**
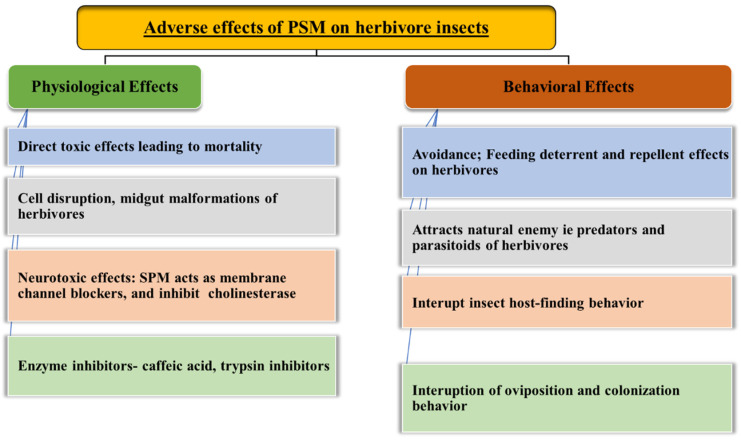
Adverse effects of PSM on the physiology and behavior of herbivores.

Plants need to respond rapidly to herbivory and produce defense chemicals at the site of the attack. Secondary metabolites may repel or deter the feeding or cause direct toxic symptoms leading to inhibition of growth which ultimately results in the death of insects (Figure 3). Recent molecular tools enable us to detect the target sites of these toxins at cellular or molecular level. Defense response of these PSM compounds is flexible which is modulated by herbivore damage [101]. Some of these metabolites are constitutive while others are generated after herbivore attacks. Induced defense metabolites are produced only after insect attack [102]. Constitutive defenses mainly rely on carbon-based metabolites, such as terpenes and polyphenols, that contribute to a great extent to the dry matter content in plants and are concentrated in special structures or compartments, such as resin canals in the xylem of coniferous trees [103], and the cell wall of cereals [104].

PSMs are bioactive compounds that repel or intoxicate insects and impair their digestion. Alkaloids are generally unfavored by insect pests and act as feeding deterrents, growth inhibitors and target neurotransmitters, affecting neuronal signal transduction [105]. Alterations in the concentrations, as well as the expression of neurotransmitters, is impaired by alkaloids. This subsequently leads to adverse physiological and behavioral changes in insects, causing direct toxic effects on insects, or insects may not prefer the specific host for feeding. Alkaloids that modulate neuronal signal transduction are nicotine, caffeine, erythrina alkaloids, tubocurarine, ergot alkaloids, muscarine, agroclavine, theophylline [106]. The alkaloid caffeine showed insecticidal properties, including paralysis and intoxication through inhibition of phosphodiesterase activity against herbivores and can be used as a potential biopesticide [107]. Nicotine, an alkaloid from tobacco, was inadvertently used for insect pest management [108]. Other secondary metabolites, such as pyrethrum from members of the Asteraceae, azadirachtin extracted from need seed, and capsaicin, obtained from extracts of hot pepper, have been used as insecticides [109]. These metabolites affect the insect in different ways, for example, blocking of receptors and channels which are involved in the nervous system, inhibition of cellular respiration and interruption of the hormonal balance of the insect [110]. 

Some PSMs act as protein inhibitors. For example, ricin, albrine, lycorine and emetine impede the process of protein synthesis in ribosomes, however, few others target protein structure and function. Some PSMs target the cytoskeleton of cells or interrupt the process of cell division. Specific inhibitor compounds, such as colchicine, sanguinarine and rotenone, inhibit mitotic cell division. Phenolic compounds interact with the protein of target organisms by forming multiple hydrogen and ionic bonds, thus modifying the 3D structure of proteins [106]. The biological parameters of the maize stalk borer, *Chilo partellus*, were affected on resistant maize germplasm implying that the antibiosis resulted because of the presence of secondary metabolites induced in the plants due to larval feeding [111]. Diet incorporation assay with phenolic compounds (ferulic and p-coumaric acids) revealed an antibiosis effect on *Sesamia inferens* larvae [112].

Some PSMs are highly reactive, with unstable functional groups that specifically target the amino, hydroxyl or sulfhydryl groups of amino acid residues of proteins, ultimately modifying their functional and structural characteristics. Some PSMs are lipophilic, including mono, sesqui, di, and triterpenes, phenylpropanoids, mustard oils and steroids. Lipophilic terpenes reorganize the 3D structure of globular proteins. These lipophilic PSMs not only attack biomembranes surrounding living cells, as well as within intracellular compartments, but also alter the fluidity and permeability of biomembranes. Some PSMs are responsible for the intercalation of DNA. PSMs with aromatic, hydrophobic and planar functional groups intercalate in the middle of nucleotide pairs, such as GC-pairs. This intercalation results in the stabilization of DNA replication, averting the activities of helicases and RNA, and impeding the transitional steps of DNA replication. Further, frameshift mutations and deletions by intercalating PSMs cause cell death [113]. DNA intercalating metabolites include protoberberine, benzophenanthridine alkaloids, such as sanguinarine and berberine [31,114], quinine, emetine, furanoquinoline alkaloids, furanocoumarins, anthraquinones and beta-carboline alkaloids [19,31]. DNA intercalating agents cause mutations and genotoxicity by directly binding to nucleotide bases and forming covalent bonds [113].

Plants produce an array of small-molecule volatile compounds, termed herbivore-induced plant volatiles (HIPVs) [115]. HIPVs include terpenoids, volatile fatty-acid derivatives, benzenoids, phenylpropanoids, and volatile amino-acid derivatives. Natural enemies of many plant pests can use HIPVs as cues to locate infested plants and, thus, find prey/hosts [116]. HIPVs are, therefore, an indirect defense mechanism, as they attract predatory or parasitic insects, which have a negative impact on the herbivores. HIPVs can also have direct effects against insect pests; for example, indole can negatively impact african cotton leafworm food consumption and survival. The terpenes zingiberene, curcumene, p-cymene, terpinene, and phellandrene from wild tomato species are repellent to whitefly (*Bemisia tabaci*) [117]. Volatile terpenes, such as 7-epizingiberene (sesquiterpene) from *Solanum habrochaites*, were reported to repel the silver leaf whitefly *Bemisia tabaci*, thus lessening the plant-insect interaction or contact, and ultimately transmission of viral disease caused by begomoviruses, such that they could be effectively managed [118]. Introgression of 7-epi-zingiberene biosynthetic pathways in tomato cultivars enabled the plant to be less attractive to whitefly and toxic to spider mites (*Tetranychus urticae*) [119]. Recently, several studies have reported the effectiveness of PSMs against herbivores applied in an eco-friendly way (Supplementary Table S1).

## 7. Adaptations of Herbivore Insects to Plant Secondary Metabolites

**Figure 4:**
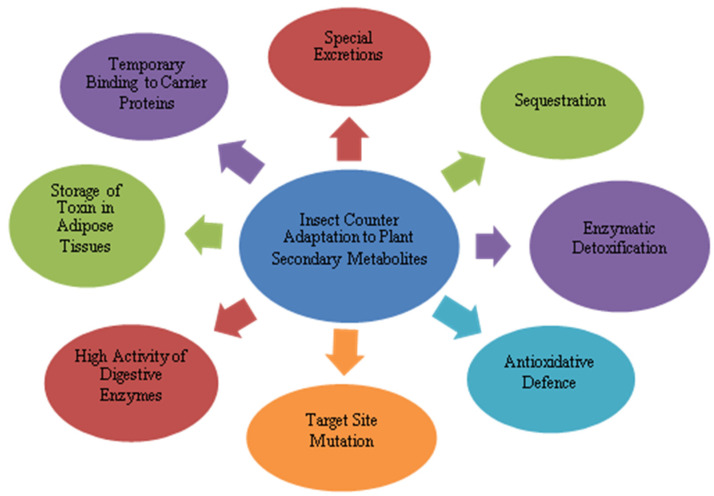
Insect Counter Adaptation to Plant Secondary Metabolites.

The continuous adaptation of insect pests to plant defensive characters is responsible for the coexistence of insects. Insect herbivores adapt themselves to plant secondary metabolites by different strategies, including by detoxifying plant toxins, alteration of the toxic compounds into favorable compounds for their growth and development, developing the choice of feeding on the basis of secondary metabolite concentration, quick engrossment and expulsion as feces, and enlisting the aid of symbiotic intestinal microbes in order to mitigate the effect of toxic PSMs [10,120] (Figure 4). Furthermore, herbivores also employ secondary metabolites as chemical indicators through their well-developed chemoreceptors. Toxic secondary metabolites are stored by insects, which are later utilized as protection against their natural enemies and to protect their eggs [120]. However, the innate ability of flora to produce chemicals in defense against insect attack should be fully exploited in order to improve the performance of secondary metabolites. Subduing the host defense response through use of secreted protein effectors is also one of the adaptive mechanisms in insects to PSMs [121].

Several factors contribute to the successful adaptation of insect herbivores, one being insect robust olfactory systems and swift evolution [122]. The excitation of specific proteins, namely odorant-binding proteins (OBPs), olfactory receptors (ORs) and gustatory receptors (GRs), help in the recognition of the chemicals [123]. The evolution of specific proteins has enabled the synchronization of genes in the insect against various stresses [124]. For example, during feeding, secondary metabolites impacting the silkworm, *Bombyx mori*, are sensed by the GR gene group which consists of special receptors [125], whereas in the case of the postman butterfly, *Heliconius melpomene*, the GR gene is involved in plant-specific oviposition [126]. Another factor is the up and/or downregulation of numerous genes encoding several enzymes. For example, the cowpea bruchid, *Callosobruchus maculatus* recognizes soybean cystatin (a cysteine protease inhibitor, scN) due to the upregulation of genes encoding proteins and carbohydrates [127]. The pattern of insect feeding habits is that they do not only feed on a single plant but, rather, they scout for a larger species with less PSM content, thereby diluting the toxicity. Phytophagous insects have a faster digestion process which aids in the more rapid absorption of nutrients than any of the toxic chemicals and thereby leads to a quick discard. The presence of symbiotic intestinal microorganisms helps in the degradation of the toxins [128]. In the case of tannins, apart from insects having adapted to them, they may also use tannins for their growth and development [129]. For example, the tree locust, *Anacridium melanorhodon*, showed an increase in growth of 15% when fed on a tannin-containing diet [130].

Plants have evolved highly impactful and energetic defense mechanisms, but all these mechanisms are exposed to insect counter-adaptation. Since the counter-adaptation of insects is highly complex, it has created challenges in the development of plant varieties resistant to insect pests. Hence, a complete understanding of herbivore insect counter-adaptations to PSMs is essential to determine insect pest management strategy.

## 8. Functional Role of PSMs in Mediating the Multi-Trophic Interactions

Plant secondary metabolites play an important role in mediating interactions with insect herbivores and their natural enemies. Insect pollinators are lured to flowers by their color or scent; color is produced by flavonoids, anthocyanins, or carotenoids, whereas terpenoids, amines, and phenylpropanoids have distinct scents that bees can distinguish. Flowers provide sugar-rich nectar as a reward for pollinators, which they prefer above other flowering materials [19].

As nectar is the major reward to pollinators, the presence of defensive metabolites in the nectar of plant species is undesirable. Recent studies have shown that PSMs can provide several benefits to both the plant and pollinators [17]. PSMs produced for plant defense against herbivores are similar to those produced in the floral nectar and pollen. This is due to the fact that chemical defenses in a single plant species are regulated by specific biochemical pathways and a particular group of secondary metabolites is produced in the plant to deliver diverse functions. The interlinkage of these metabolites for floral rewards and plant defense is not well understood. Phytochemicals involved in plant defense and attraction of pollinators include caffeine, aconitine, nicotine, thymol, linalool, lupanine and grayanotoxins [17]. Investigations into the effects of caffeine on bees revealed surprising results. Caffeine is present in the floral nectar of *Citrus* spp. and *Coffea* spp. Caffeine initially acts as a feeding deterrent to honeybees in a dose-dependent manner. However, the caffeine levels present in the nectar were below the bees’ taste detection limit. At a naturally occurring level, caffeine restimulated the memory of bees and enhanced their ability to find a nectar source using floral scent which ultimately improved the olfactory learning and memory of honeybees [131]. PSMs present in the flower nectar and pollen can help bees to overcome external bee pathogens and reduce their transmission. They can be actively effective against internal gut parasites of bees and lessen the pathogen infection load in foraging bees [132]. Several phytochemicals occurring in nectar, such as alkaloids, terpenoids and iridoid glycosides, are effective in reducing the intestinal parasite, *Crithidia bombi*, in eastern bumblebees, *Bombus* spp. [133]. Insect herbivory, including root herbivory, folivory, and florivory, has been shown to alter floral characteristics, such as size, pollen content, and nectar volume [134,135,136]. Changes in flower characteristics can have a direct impact on pollinator behavior. Focused research is required on the regulation of floral metabolites in plants to comprehend how secondary metabolites are involved in plant defensive and pollinator-supporting roles.

PSMs modulate the behavior, predation, parasitism and physiology of natural enemies of herbivores. Volatiles emitted after herbivore injury are utilized to locate host insects by herbivore predators and parasitoids [116,137]. These metabolites adversely affect the effectiveness of natural enemies, when they are transferred through herbivores that selectively accumulate and sequester these metabolites for self-defense [138]. Recent evidence suggests the development of resistance or tolerance to cardenolides and glucosinolates in different predators [139]. Resistance in natural enemies was quantified by manipulation of the synthesis or processing of PSMs. Silencing glucosinolate sulfatase to increase toxic isothiocyanate accumulation in diamondback moth, *Plutella xylostella*, larvae, enables the predatory lacewing, *Chrysoperla carnea*, to grow slowly without any adverse effects on its biology [140]. Predators and parasitoids can develop resistance to PSMs when preying on adapted specialist herbivores. This resistance is accompanied with improved fitness and predatory potential. PSMs have the potential to augment or hamper biological control through serving as host locating cues or suppressing the performance of natural enemies [141].

Extended herbivore development is likely to increase the exposure duration of herbivores to predation and parasitism [142]. As parasitoid attacks a specific developmental stage of a host, prolongation of a vulnerable growth stage can increase parasitism, though this affects predation less, if predators consume a broad range of prey and are not stage-specific. The slow growth-high mortality hypothesis was evaluated empirically, and increased parasitism was observed for herbivore hosts that grow more slowly [143]. Linalool acts as a deterrent compound to the rice brown planthopper (*Nilaparvata lugens*) in rice and attracts the natural enemies of *N. lugens* [144]. However, the role of a single compound in the deterrence of herbivores and attraction of natural enemies is an unresolved mystery.

Phytochemicals from ribwort plantain, *Plantago lanceolate*, stimulate the accelerated growth of the specialist herbivore, *Melitaea cinxia*, and its solitary endoparasitoid, *Hyposoter horticola*, when reared on a high iridoid glycosides line. Conversely, the pupal weight of the generalist herbivore, *Spodoptera exigua*, and the adult mass of its solitary endoparasitoid, *Cotesia marginiventris*, was significantly reduced when reared on a high iridoid line [145]. Several adverse effects on the biological parameters of *Bracon hebetor* were reported, when its host insect, *Spodoptera litura*, was exposed to phytochemicals from *Inula racemose*. Reduced parasitism, decreased survival and prolonged development of the Braconid parasitoid were observed [146]. The adverse effects of PSMs on natural enemies enable us to think about the manipulation of PSMs for enhanced plant protection and aid in comprehending how PSMs influence multitrophic interactions under natural conditions.

## 9. Commercial Production of Secondary Metabolites: Barriers and Biotechnological Prospects

Increasing global food production while improving crop quality and minimizing the environmental impact of agricultural practices is becoming one of the potential difficulties in achieving food security [147]. To mitigate the challenge of herbivore insects sustainably, restricted usage of synthetic pesticides and promotion of the usage of environment- friendly, plant-based pesticides are needed. Secondary plant metabolites can serve as alternatives to synthetic pesticides to stimulate plant growth and plant health [148]. Several phytochemicals were screened in search of potential pest control products worldwide. The market of biopesticides is rising at 16% per annum in comparison to conventional pesticides which are growing at 5.5% per year [81]. However, major roadblocks to the commercialization of plant-based pesticides include: (a) botanical resource accessibility and sustainability; (b) strength, standardization, and quality control of the chemically composite extracts based on quantification of active ingredients; and (c) regulatory support, which usually necessitates a costly toxicological assessment of the potential product [27].

Generally, PSMs can be extracted from naturally available plants, but large-scale production is restricted due to geographical and environmental variations [149]. Traditional methods are time-consuming, as the plant takes several years to grow and reach the desired level of metabolite synthesis. Traditional approaches rely on the rates at which substrates from primary metabolic pathways are re-routed to secondary biosynthetic pathways. PSM biosynthesis is governed by stress factors, such as temperature, humidity, light intensity, herbivore injury or pathogen attack, growth and physiology [150]. Enhanced production of PSMs is achieved by using elicitors which trigger secondary metabolic pathways to activate plant defense mechanisms [151]. Phytohormones, such as salicylic acid and methyl jasmonate, under biotic stress conditions are reported to be the signaling molecules in elicitation and enhanced synthesis of PSMs, such as flavonoids, alkaloids, terpenoids and phenylpropanoids [152]. Nowadays, plant cell tissue culture and metabolic engineering are novel techniques available for the commercial production of PSMs in a short period [153,154]. Both these techniques allow large-scale propagation of plants in protected environmental conditions irrespective of the season. In the plant tissue culture technique, a two-stage procedure is involved under in vitro conditions: (a) aggregation of biomass, and (b) synthesis of PSMs [149]. Organized structures, such as shoots and roots, callus, cell suspensions, etc., are used for the large-scale production of PSMs [155]. Metabolic engineering is another attractive and novel approach in which alterations of metabolic routes are performed using biotechnological tools, such as genomics, proteomics, metabolomics, etc., to enhance the production of PSMs [153]. It allows manipulation of endogenous biosynthetic pathways in plants involving up or downregulation of metabolite pathways by averting the precursors, enzymes, and regulatory proteins using recombinant DNA technology [156].

Novel approaches, which consider the behavior and controlled release of individual components of botanicals, are needed to create a new generation of highly potent and efficient botanical pesticides. PSMs can be used as effective pesticides only when the production hurdles/limitations are overcome using novel biotechnological tools, such as metabolic engineering and plant tissue culture. Plant-derived products cannot supersede conventional synthetic pesticides, but these products should be favored in regulatory procedures where special attention is given to environmental safety.

## 10. Role of Plant Secondary Metabolites in Sustainable Crop Protection

Agro-ecosystems suffer from insect pests, which adversely affect crop production. The most effective means for managing pests is to use synthetic pesticides, which are easy to use and are readily available to farmers [157]. The adverse effects of these agrochemicals on the environment and human health [158], as well as pesticide-resistant pests [159], are promoting the need for sustainable pest control. Plant secondary metabolites, which have low toxicity to humans and the environment, with multiple mechanisms of action, could be a viable alternative to pesticides in sustainable agriculture. Their varied modes of action are attributed to the phytochemical composition in different plants.

PSMs, such as volatile compounds, can attract pollinators and they also play a vital role in the direct and indirect defenses of plants [160]. For example, tobacco plants (*Nicotina attenuata*) suppress nicotine production when attacked by the tobacco hornworm (*Manduca sexta*), a nicotine-tolerant folivore and, instead, emit volatile organic compounds [E-bergamotene] that attract the generalist predator *Geocoris pallens* as a defense against *M. sexta* [161]. Crop and forage varieties that are improved to enhance PSM production could eliminate the need for synthetic insecticides. Nicotine, for example, has been successfully used as an insecticide because it repels herbivores [162]. Pyrethrins and the triterpene azadirachtin are two other insecticidal PSMs that are least toxic to non-target organisms yet act as effective insect deterrents. Pyrethroids are the most important natural biochemicals used to develop synthetic pesticides because they are extremely efficient at repelling insects, leave minimal pesticide residues and are safe to mammals [163]. Secondary metabolites form an integral part of sustainable agriculture and have no negative environmental consequences. PSMs have the potential to aid in the conservation of natural resources and the elimination of problems caused by the application of chemicals. They can also be used to save the crop in a cost-effective and ecologically sustainable manner.

Integrated pest management is a dynamic and constantly evolving approach to crop protection in which all the suitable management tactics and available surveillance information are utilized to develop a holistic management program. Sustainable pest management aims to minimize the destruction of the natural environment and achieve a high and profitable yield [164]. PSMs are natural products that are effective against insect pests. They are highly biodegradable, have varied modes of action, are less toxic to humans, are non-pollutant and are readily available in the environment [165]. Thus, PSMs have the potential to be a part of IPM, along with other crop protection techniques, such as host plant resistance, good agricultural practices, the use of natural enemies, such as predators and parasitoids, microbial pesticides and the judicious use of selective synthetic pesticides. This strategy, together with early pest detection and monitoring, would result in quick, effective and sustainable crop protection against herbivores [166].

## 11. Conclusions and Future Prospects

Secondary metabolites have become a subject of great interest because of their significant application in plant stress physiology. These metabolites can play a promising role in maintaining the health and productivity of food crops, even under stressful conditions, with minimum loss. Thus, manipulation and overexpression of PSM biosynthesis pathway-related genes could be a solution to combat herbivore attack and injuries. PSMs mediate a variety of defensive functions by elevating the synthesis of various enzymes responsible for secondary metabolite production and enhancing the expression of various genes involved in resistance mechanisms of plants. Defense-related secondary metabolite biosynthesis is regulated by plants’ early defense signaling. Therefore, there is broad scope in the future to shed more light on the molecular regulation of herbivore-mediated secondary metabolite biosynthesis for the advancement of insect-resistant traits in crop plants. Biotechnological tools, such as in vitro plant cell culture and metabolic engineering, can be used effectively for the production of secondary metabolites. PSMs modulate the behavior, predation, parasitism and physiology of natural enemies of herbivores. Crop domestication could weaken plant defenses, lowering intrinsic resilience to herbivory and this could have an impact on secondary metabolites in nectar and pollen, influencing pollinators. Plant defense mechanisms are overcome by herbivore insects through counter-adaptations. Hence thorough research into plant-herbivore interactions is essential to devise pest management strategy. There is scope to investigate unidentified plant secondary metabolites from the large wild gene pool of plants, and to evaluate their repellent and deterrent effects against herbivores. Emerging molecular genetic approaches have tremendous potential to unravel the regulatory genes that control plant secondary metabolite biosynthesis. This information, together with increased knowledge of the enzymes specific to the pathways, could facilitate the genetic engineering of plants.

## Figures and Tables

**Table 1:** External application of phytohormones in inducing plant defense to herbivores.

Type of Insect	Plant Species	Induced Defense	Protective Function	Reference
**Effect of JA and Its Derivatives**				
*Helicoverpa armigera*	*Arachis hypogaea*	Increased activity of POD, PPO and total phenol, H_2_O_2_ and MDA	Host plant defense to herbivore	[87]
*Pieris rapae* and *Plutella xylostella*	*Brassica oleracea*	Emission of volatile compounds, such as β-ocimene, thuja 2,4(10)-diene, and terpinene	Attraction of parasitoids, such as *Cotesia glomerata*, *C. rubecula*, and *Diadegma semiclausum*	[88]
*Nilaparvata lugens*	Rice	Emission of plant volatiles, i.e., aliphatic aldehydes, alcohols, monoterpenes, sesquiterpenes, methyl salicylate, etc.	Attracts *Anagrus nilaparvatae* (egg parasitoid)	[89]
*Diatraea saccharalis*	Sugarcane	Emission of blend of sesquiterpenes	Attracts parasitoid *Cotesia flavipes*	[90]
*Plutella xylostella*	*Brassica napus*	Production of glucosinolates and trypsin inhibitor	Reduced survivorship of *Plutella xylostella*	[91]
*Spider mites*	Lima bean (*Phaseolus lunatus*)	Transcript levels of (E)-β-ocimene synthase (PlOS) increased and increased emission of (E)-β-ocimene	Enhanced biological control of spider mites due to increased volatiles emission	[92]
**Effect of SA and Its Derivatives**				
*Frankliniella occidentalis*	*Jacobaea. aquatica*	Increased levels of threonine, citric acid, and alanine	Reduced thrips population and inhibited feeding	[93]
*Oebalus pugnax*	Rice, *Oryza sativa*	Increased plant volatiles production	Reduced damage by stinkbug and prevented formation of spikelet sterility	[94]
Aphids/mites	Washington hop yard	Indirect defense	More attraction of *Chrysopa nigricornis*	[95]
*Bactrocera dorsalis*	Mango	Increased levels of anti-oxidative enzymes, such as catalase, peroxidase, poly phenoloxidase, along with phenol and flavonoid	Reduced oviposition, larval and adult emergence of *Bactrocera dorsalis*	[96]
*Helicoverpa armigera*	Ground nut	Increased glutathione s-transferase activity	Reduction in larval weight and survival	[87]
*Psyllid, Agonoscena pistaciae*	Pistachio	High phenol and H_2_O_2_production	Reduced survival of *A. pistaciae*	[97]
*Psyllid, Agonoscena pistaciae*	*Pistacia vera*	Increased production of anti-oxidative enzymes, such as polypheloxidase, and peroxidase	Reduction in the number of eggs and in nymphal density	[98]
**Effect of Gibberellic Acid(GA)**				
*Spodoptera frugiperda*	*Maize*	Increased silicon uptake in plants	Decreased fecundity and reduced feeding on corn in *S. frugiperda*	[99]
*Pea aphid, Acyrthosiphon pisum*	*Medicago truncatula*	Increased levels of JA and SA and JA-related plant gene expression	Decreased fitness of *A. pisum*	[100]

POD: peroxidase; PPO: polyphenol oxidase; H_2_O_2_: hydrogen peroxide; MDA: malondialdehyde.

## Supplementary Materials

The following supporting information can be downloaded at: https://www.mdpi.com/article/10.3390/ijms23052690/s1 [162,167,168,169,170,171,172,173,174,175,176,177,178,179,180,181,182,183,184,185,186,187,188,189,190,191,192,193,194].
