# Supplementary material for: Plant Secondary Metabolites as Defense Tools against Herbivores for Sustainable Crop Protection
Source: Int J Mol Sci. 2022 Feb 28;23(5):2690. doi: 10.3390/ijms23052690 (PMC8910576; doi:10.3390/ijms23052690)
Supplement: Supplementary file 1 [file ijms-23-02690-s001.zip › ijms-1559780-supplementary.pdf]

Supplementary Table S1. Mode of action of plant secondary metabolites against herbivores

**Secondary Metabolites Type**	**SPM**	**Target Insect**	**Adverse Effects**	**Reference**
**Repellents**				
Sesquiterperne	(E)-β-caryophyllene	Asian citrus psyllid *Diaphorina citri*	Repellent and feeding deterrent deterrence	[3]
Alkaloids	Nicotine	*Diabrotica undecimpunctata*, *Spodoptera exigua* and *Trimerotropis* spp.	Repellent and deterrence in feeding	[25]
Alkaloids	α-solanine and α-chaconine	Guatemalan potato moth (*Tecia solanivora*)	Poor oviposition due to repellent effect and reduced larval survival	[15]
Essential oil	Patchoulol	red flour beetle , *Tribolium castaneum*; cigarette beetle *Lasioderma serricorne* and booklice *Liposcelis bostrychophila*	Repellent effects	[13]
Sesquiterpene,	(S)-germacrene	cereal aphid, *Sitobion avenae*	Repellent effects	[6]
Essential oils	-	red flour beetle, *Tribolium castaneum*	Repellent effects	[20]
Phenolics	ginkgo flavonoids, ginkgolide, and bilobalide	*Hyphantria cunea*	Strong antifeedant and repelling effects	[21]
Annonaceous acetogenins	squamocin	*Spodoptera frugiperda*	Toxic and lethal effects on early instar larvae	[8]
**Feeding Deterrents**				
Saponin	saponin-aglycones hederagenin cellobioside and oleanolic-acid cellobioside	Diamondback moth (*Plutella xylostella*)	Proved as effective feeding deterrents	[1,4,24]
Phenolics	Rutin, vanillic and synaptic acid	Oriental leafworm *Spodoptera litura*	Adversely affected the larval growth and development	[19]
Mustard oils	Isothiocyanates	*Pieris rapae*	Reduced survival and growth and prolong the larval development	[2]
	cyanogenic glycosides and dhurrin	Flea beetle (*Phyllotreta nemorum*)	Reduced the larval survival	[27]
Phenolics	Chlorogenic acid	*Spodoptera litura*	Inhibits the larval growth of all the instars	[17]

Phenolics	Furanocoumarin, 5-(6-hydroxy-3,7-dimethylocta-2,7-dienyloxy) psoralen	*Spodoptera littoralis* and *Heliothis virescens*	Feeding deterrent effect towards the larvae	[26]
	Catechins, scopoletin, cedrelone	*S. frugiperda,*	Reduced the larval survival of larval weight	[14]
	Aglaroxin A	Gram pod borer, *Helicoverpa armigera* and Asian armyworm, *Spodoptera litura*	Strong antifeedant properties and reduction in growth of the larvae of	[16]
	Fraxinellone	*Mythimna separata*	Exhibited toxic effects in terms of prolonged and reduced egg hatching, inhibition of growth and pupation	[18]
	Rhodojaponin-III	*Spodoptera litura* moths	Oviposition deterrent	[29]
**Growth Inhibitory Effects**				
	1′-acetoxychavicol acetate and galangin	*Spodoptera litura*	Inhibitory effects on various nutritional parameters of	[10]
	1′-acetoxychavicol acetate and galangin	*Spodoptera litura*	Exhibited more larvicidal activity and retarded the larval development	[9]
	Tocotrienols and hydroquinones	*Spodoptera frugiperda*	Growth inhibitory and lethal effects on the larvae	[7]
	Ononitol monohydrate	*Helicoverpa armigera* and *Spodoptera litura*	Strong antifeedant, larvicidal and growth inhibitory activities. Larval and pupal periods were prolonged	[5]
	Limonoid Khayanolide B	*Spodoptera littoralis*	Potent antifeedant compound showed remarkable inhibition of larval growth and development	[12]
	Triterpenoid saponins, monodesmosides 1, 2, and 5	*Plutella xylostella*	Reduction of larval growth and percentage of pupation. Prolongation of larval and pupal stages	[28]
	flavonoid, quercetin	*Spodoptera litura*	94% larval mortality and significantly reduced larval growth of *Spodoptera litura*	[22]
	Gallic acid	Melon fruit fly, *Bactrocera cucurbitae*	Larval survival and adult emergence were severely affected. Inhibitory effects	[23]

			of gallic acid were also observed on larval weight, pupal weight and mean relative growth rate	
	Rolliniastatin-1	*Helicoverpa armigera*	Increased larval mortality and significantly decreased foliar consumption. Significantly decrease in the growth rate and prolonged larval duration along with reduced viability of the larval and pupal stages and reduced larval weight	[11]

References:

1. Agerbirk, N.; Olsen, C.E.; Bibby, B.M.; Frandsen, H.O.; Brown, L.D.; Nielsen, J.K.; Renwick, J.A.A. A saponin correlated with variable resistance of Barbarea vulgaris to the diamondback moth *Plutella xylostella*. *J. Chem. Ecol.* **2003**, *29*, 1417-1433.
2. Agrawal, A.A.; Kurashige, N.S. A role for isothiocyanates in plant resistance against the specialist herbivore *Pieris rapae*. *J. Chem. Ecol.* **2003**, *29*, 1403-1415.
3. Alquézar, B.; Volpe, H.X.L.; Magnani, R.F.; de Miranda, M.P.; Santos, M.A.; Wulff, N.A.; Peña, L. β-caryophyllene emitted from a transgenic Arabidopsis or chemical dispenser repels *Diaphorina citri*, vector of *Candidatus Liberibacters*. *Sci. Rep.* **2017**, *7*, 1-9.
4. Badenes-Perez, F.R.; Gershenzon, J.; Heckel, D.G. Insect attraction versus plant defense: young leaves high in glucosinolates stimulate oviposition by a specialist herbivore despite poor larval survival due to high saponin content. *PLoS One* **2014**, *9*, e95766.
5. Baskar, K.; Ignacimuthu, S. Antifeedant, larvicidal and growth inhibitory effects of ononitol monohydrate isolated from *Cassia tora* L. against *Helicoverpa armigera* (Hub.) and *Spodoptera litura* (Fab.) (Lepidoptera: Noctuidae). *Chemosphere* **2012**, *88*, 384-388.
6. Birkett, M.A.; Bruce, T.J.; Pickett, J.A. Repellent activity of *Nepeta grandiflora* and *Nepeta clarkei* (Lamiaceae) against the cereal aphid, *Sitobion avenae* (Homoptera: Aphididae). *Phytochem. Lett.* **2010**, *3*, 139-142.
7. Céspedes, C.L.; Torres, P.; Marın, J.C.; Arciniegas, A.; de Vivar, A.R.; Pérez-Castorena, A. L.; Aranda, E. Insect growth inhibition by tocotrienols and hydroquinones from *Roldana barbajohannis*. *Phytochemistry* **2004**, *65*, 1963-1975.
8. Colom, O.Á.; Neske, A.; Popich, S.; Bardón, A. Toxic effects of annonaceous acetogenins from *Annona cherimolia* (Magnoliales: Annonaceae) on *Spodoptera frugiperda* (Lepidoptera: Noctuidae). *J. Pest Sci.* **2007**, *80*, 63-67.

9. Datta, R.; Kaur, A.; Saraf, I.; Kaur, M.; Singh, I.P.; Chadha, P.; Kaur, S. Assessment of genotoxic and biochemical effects of purified compounds of *Alpinia galanga* on a polyphagous lepidopteran pest *Spodoptera litura* (Fabricius). *Phytoparasitica* **2020**, *48*, 501-511.
10. Datta, R.; Kaur, A.; Saraf, I.; Singh, I.P.; Kaur, S. Effect of crude extracts and purified compounds of *Alpinia galanga* on nutritional physiology of a polyphagous lepidopteran pest, *Spodoptera litura* (Fabricius). *Ecotoxicol. Environ. Saf.* **2019**, *168*, 324-329.
11. de Souza, C.M.; Baldin, E.L.L.; do Prado Ribeiro, L.; dos Santos, T.L.B.; da Silva, I.F.; Morando, R.; Vendramim, J.D. Antifeedant and growth inhibitory effects of Annonaceae derivatives on *Helicoverpa armigera* (Hübner). *Crop Prot.* **2019**, *121*, 45-50.
12. El-Aswad, A.F.; Abdelgaleil, S.A., Nakatani, M. Feeding deterrent and growth inhibitory properties of limonoids from *Khaya senegalensis* against the cotton leafworm, *Spodoptera littoralis*. *Pest Manag. Sci.* **2004**, *60*, 199-203.
13. Feng, Y.X.; Wang, Y.; You, C.X.; Guo, S.S.; Du, Y.S.; Du, S.S. Bioactivities of patchoulol and phloroacetophenone from *Pogostemon cablin* essential oil against three insects. *Int. J. Food Prop.* **2019**, *22*, 1365-1374.
14. Giongo, A.M.M.; Vendramim, J.D.; Freitas, S.D.L.; Silva, M.F.G.F. Toxicity of secondary metabolites from Meliaceae against *Spodoptera frugiperda* (JE Smith) (Lepidoptera: Noctuidae). *Neotrop. Entomol.* **2016**, *45*, 725-733.
15. Karlsson, M.F.; Birgersson, G.; Witzgall, P.; Lekfeldt, J.D.S.; Punyasiri, P.N.; Bengtsson, M. Guatemalan potato moth *Tecia solanivora* distinguish odour profiles from qualitatively different potatoes *Solanum tuberosum* L. *Phytochemistry* **2013**, *85*, 72-81.
16. Koul, O.; Singh, G.; Singh, R.; Multani, J.S. Bioefficacy and mode-of-action of aglaroxin A from *Aglaia elaeagnoidea* (syn. *A. roxburghiana*) against *Helicoverpa armigera* and *Spodoptera litura*. *Entomol. Exp. App.* **2005**, *114*, 197-204.
17. Kundu, A.; Mishra, S.; Vadassery, J. *Spodoptera litura*-mediated chemical defense is differentially modulated in older and younger systemic leaves of *Solanum lycopersicum*. *Planta* **2018**, *248*, 981-997.
18. Lv, M.; Wu, W.; Liu, H. Effects of fraxinellone on the midgut enzyme activities of the 5th instar larvae of oriental armyworm, *Mythimna separata* walker. *Toxins* **2014**, *6*, 2708-2718.
19. Movva, V.; Pathipati, U.R. Feeding-induced phenol production in *Capsicum annuum* L. influences *Spodoptera litura* F. larval growth and physiology. *Arch. Insect Biochem. Physiol.* **2017**, *95*, e21387.

20. Olivero-Verbel, J.; Tirado-Ballestas, I.; Caballero-Gallardo, K.; Stashenko, E.E. Essential oils applied to the food act as repellents toward *Tribolium castaneum*. *J. Stored Prod. Res.* **2013**, *55*, 145-147.
21. Pan, L.; Ren, L.; Chen, F.; Feng, Y.; Luo, Y. Antifeedant activity of *Ginkgo biloba* secondary metabolites against *Hyphantria cunea* larvae: mechanisms and applications. *PLoS One* **2016**, *11*, e0155682.
22. Selin-Rani, S.; Senthil-Nathan, S.; Thanigaivel, A.; Vasantha-Srinivasan, P.; Edwin, E.S.; Ponsankar, A.; Alessandro, R.T. Toxicity and physiological effect of quercetin on generalist herbivore, *Spodoptera litura* Fab. and a non-target earthworm *Eisenia fetida* Savigny. *Chemosphere* **2016**, *165*, 257-267.
23. Sharma, R.; Sohal, S.K. Bioefficacy of quercetin against melon fruit fly. *Bull. Insectology* **2013**, *66*, 79-83.
24. Shinoda, T.; Nagao, T.; Nakayama, M.; Serizawa, H.; Koshioka, M.; Okabe, H.; Kawai, A. Identification of a triterpenoid saponin from a crucifer, *Barbarea vulgaris*, as a feeding deterrent to the diamondback moth, *Plutella xylostella*. *J. Chem. Ecol.* **2002**, *28*, 587-599.
25. Steppuhn, A.; Gase, K.; Krock, B.; Halitschke, R.; Baldwin, I.T.; Levine, M. Nicotine's defensive function in nature. *PLoS Biol.* **2004**, *2*, e217.
26. Stevenson, P.C.; Simmonds, M.S.; Yule, M.A.; Veitch, N.C.; Kite, G.C.; Irwin, D.; Legg, M. Insect antifeedant furanocoumarins from *Tetradium daniellii*. *Phytochemistry* **2003**, *63*, 41-46.
27. Tattersall, D.B.; Bak, S.; Jones, P.R.; Olsen, C.E.; Nielsen, J.K.; Hansen, M.L.; Møller, B.L. Resistance to an herbivore through engineered cyanogenic glucoside synthesis. *Science* **2001**, *293*, 1826-1828.
28. Tian, X.; Li, Y.; Hao, N.; Su, X.; Du, J.; Hu, J.; Tian, X. The antifeedant, insecticidal and insect growth inhibitory activities of triterpenoid saponins from *Clematis aethusifolia* Turcz against *Plutella xylostella* (L.). *Pest Manag. Sci.* **2021**, *77*, 455-463.
29. Yi, X.; Liu, J.; Wang, P.; Hu, M.; Zhong, G. Contacting is essential for oviposition deterrence of rhodojaponin-iii in *Spodoptera litura*. *Arch. Insect Biochem. Physiol.* **2014**, *86*, 122-136.
